# Nanomedicine in Non-Small Cell Lung Cancer: From Conventional Treatments to Immunotherapy

**DOI:** 10.3390/cancers12061609

**Published:** 2020-06-18

**Authors:** Coral García-Fernández, Cristina Fornaguera, Salvador Borrós

**Affiliations:** Grup d’Enginyeria de Materials (GEMAT), Institut Químic de Sarrià (IQS), Universitat Ramon Llull (URL), 08022 Barcelona, Spain; coralgarciaf@iqs.url.edu (C.G.-F.); salvador.borros@iqs.url.edu (S.B.)

**Keywords:** non-small cell lung cancer, immunotherapies, nanomedicine, cancer vaccine, chemotherapy

## Abstract

Non-small cell lung cancer (NSCLC) remains the most common cause of cancer-related mortality. The heterogeneous nature of this disease hinders its diagnosis and treatment, requiring continuous advances in research aiming to understand its intricate nature. Consequently, the retrospective analysis of conventional therapies has allowed the introduction of novel tools provided by nanotechnology, leading to considerable improvements in clinical outcomes. Furthermore, the development of novel immunotherapies based on the recently understood interaction of the immune system with the tumor highlights the real possibility of definitively treating NSCLC from its early stages. Novel engineering approaches in nanomedicine will enable to overcome the intrinsic limits of conventional and emerging therapies regarding off-site cytotoxicity, specificity, resistance mechanisms, and administration issues. The convergence point of these therapies with nanotechnology lays the foundation for achieving currently unmet needs.

## 1. Introduction

Lung cancer is the major cause of cancer death and one of the leading causes of death worldwide. In 2018, it accounted for more than 2 million deaths, according to the data reported by the World Health Organization (WHO) [1]. Furthermore, statistical studies predict the doubling of its prevalence in the coming years. The American Cancer Society estimates more than 130,000 deaths only in the United States during 2020 [1,2]. A prospective evaluation of the incidence trend suggests that smoking habits are closely related to the appearance of lung cancer, although other environmental and genetic factors are also determinant. A remarkable increase of lung cancer cases in women has been noticed due to the rise in the number of smokers as a result of social changes, whereas the number of male smokers has traditionally been high [3,4]. Furthermore, patients suffering from this type of cancer are considered to be at high risk of worse outcomes when suffering from other respiratory conditions, such as chronic obstructive pulmonary disease (COPD) and severe acute respiratory syndrome-coronavirus-2 (SARS-CoV-2), leading to a higher number of indirect deaths [5,6,7,8].

**Figure 1:**
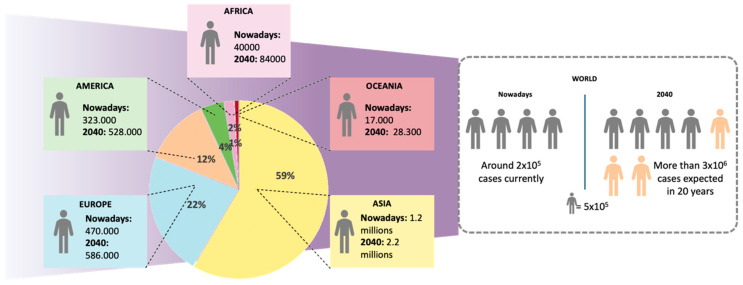
Lung cancer statistics nowadays. Geographical distribution of lung cancer cases and estimated number of cases worldwide in 2018 and 2040 (Source: Globocan).

Early diagnosis is a must for providing appropriate prognosis and treatment options, thus explaining the importance of correct lung cancer evaluation. The first classification of lung cancer was provided at the beginning of the last century [9] and has been continuously modified to respond to the increase in the number of cases as well as to the identification of new subgroups of cell lung cancer [10]. Currently, the WHO differentiates several types of lung cancer which can be mainly classified into Small Cell Lung Cancer (SCLC) and Non-Small Cell Lung Cancer (NSCLC) (Figure 1). SCLC is diagnosed in the older population, current, former, or second-hand smokers and represents approximately 20% of all cases of lung cancer [11]. NSCLC affects a much broader range of the population and accounts for 80% of all lung cancer cases. This type of cancer includes adenocarcinoma, squamous cell carcinoma, and different histotypes of large cell carcinoma (Table 1) [12,13,14]. Besides tumor classification, the procedure to be followed is decided based on the accurate staging of lung cancer, which largely depends on the histological and genetic characteristics of the patient [10,13,15]. The actual staging system is based on Tumor–Node–Metastasis (TNM) evaluation, regarding the size of the primary tumor, the spreading of cancer cells to the lymph nodes, and the tumor metastatic capacity [10,16] (Table 2). Considering metastasis is crucial for NSCLC patients, since approximately 30–40% of patients show tumor migration to other organs, most commonly the bones or the brain [17,18].

Unfortunately, often lung cancer is diagnosed at stage III or IV, or even later, owing to the absence of clinical symptoms in early stages. Therefore, it is necessary to continuously monitor those patients at risk of developing lung cancer. In advanced stages, continuous cough, chest pain, or weight loss appear, considerably reducing the quality and life expectancy of the patient [12,22,23]. Imaging techniques such as radiographs, CT, MRI, or PET (Table A1) allow to locate the tumor and to subsequently perform a biopsy of the identified area and make a diagnosis. Other techniques such as bronchoscopy, mediastinoscopy, or bronchoalveolar lung fluid analysis by bronchoalveolar lavage (BAL) (Box 1) may be necessary to complement the results [12].

**Figure 2:**
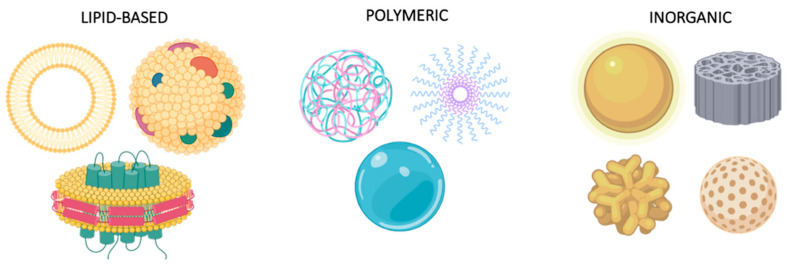
Common Nano Drug Delivery Systems. Nanoparticles can be classified into three main groups: lipidic particles, including liposomes, exosomes, and solid lipid disks among others, polymeric carriers, describing a wide range of vehicles such as polyplexes, micelles, and hydrogels, and inorganic nanosystems, the most heterogenous group, characterized by a variety of components (gold nanoparticles and stars, silica discs and spheres) and applications.

The correct prognosis and characterization of the tumor allows selecting the most effective treatment for the patient. Depending on the stage, histological cell type, and clinical condition, a wide variety of treatments are available. In the case of early diagnosis, surgical removal of the tumor, as well as radiotherapy and chemotherapy, are the chosen treatments [12,14,24]. However, despite the good short-term results, conventional treatments show a low long-term response rate due to their lack of specificity on the tumor and high off-target cytotoxicity. When locally administered, they show meaningful systematic exposure and diffuse to other tissues, which decreases their efficacy [25]. Looking for better local retention and safer systemic administration is highly desirable. To this end, a wide variety of nano Drug Delivery Systems (DDS), including liposomes, protein-based nanocarriers, inorganic carriers, and polymer nanoparticles, is being explored (Figure 2). The use of customized nanoparticles addressed to cancer cells is the next-generation approach to improve non-specific conventional therapies [26,27,28,29,30]. The preferential accumulation of nanomedicines in target tissues is commonly referred to as the Enhanced Permeability and Retention (EPR) effect [31,32]. EPR relies on the presence of abnormally wide gaps in the blood vessels surrounding a tumor. However, the failure of the passive tumor targeting of nanoparticles has questioned the clinical relevance of this phenomenon. Although a rapid tumor growth in animal models leads to the formation of blood vessels with fenestrations, this effect is not proven in human patients. Thus, researchers are now moving from passively targeted nanoparticles to actively targeted ones, addressing them to specific cell populations, like dendritic cells, in some promising immunotherapies [31,32]. However, the ability to target nanoparticles to a specific cell population is not clearly improved. Active targeting should consider the interaction of nanoparticles with proteins in the human body. Proteins adhere to nanoparticles’ surface, leading to the well-described protein corona effect and hiding the targeting molecules of the nanoparticles. This difficulty, added to recent findings on internalization mechanisms of nanoparticles in solid tumors, highlights the need to better understand the biodistribution of these carriers in the body [33].

Nanoparticle functionalization, as well as their geometry and materials, largely determine their function and, therefore, the therapeutic outcomes [31]. Furthermore, by modifying these features it is possible to use them as contrast agents for PET or CT imaging techniques, thus developing dual theragnostic platforms [34].

Regardless of the enhancements introduced by nanotools to conventional treatments (Table 3 and Table 4), more advanced tumors require combined chemotherapy and radiotherapy as standard of care, in addition to other emerging therapies such as immunotherapy or personalized medicine [10,32,35,36]. The use of immunotherapy aims to teach a patient’s immune system to recognize and eliminate tumor cells, developing a long-term anti-tumor memory of the system [12]. The development of NSCLC vaccines has arisen in recent years, supported by the design of nanoparticles specifically addressed against a cell population, such as dendritic cells [37], to awake a controlled immune response against the tumor. Despite the high expectations, there are still many challenges to be overcome due to the heterogeneity of NSCLC. Differences in overexpressed receptors in NSCLC cells in different patients explain the low percentages of response to this type of therapy [12,36]. Likewise, certain mutations in the genes that encode receptors involved in proliferation and apoptotic mechanisms, such as EFGR, ALK, ROS 1, and those related to the PI3K/Akt/mTOR pathway, can be effectively targeted by drugs inhibiting signaling cascades regulated by these receptors [10,12,32,38,39,40].

This review aims to discuss different available strategies for the treatment of NSCLC. We will analyze conventional therapies, focusing on the role of chemotherapy and molecular targeted therapies. Furthermore, we will describe emerging immunotherapies, including the design of different vaccines and gene-modulating therapies for the treatment of NSCLC. Lastly, we will provide a complete study of the current therapies for NSCLC and a further understanding of nanomedicine in this field.

## 2. Conventional Treatments

Treatments must be personalized considering a patient’s clinical condition, disease status, and tumor histological cell type. Surgery, chemotherapy, and radiotherapy have long been the preferred local treatments for NSCLC [12,24,35].

### 2.1. Surgery and Radiotherapy

Surgery is the main treatment option when NSCLC is diagnosed in the early stages I, II, and IIIA- and the patient can tolerate it. However, only 15–20% of tumors can be radically resected, because the tumor is not always clearly identifiable. During surgery, the lobe or the section of the lung containing the tumor is removed, using imaging techniques such as video-assisted thoracoscopic surgery (VATS) and biopsies as support [41,42,43,44,45]. Typically, surgery is combined with radiation therapy sessions as a neoadjuvant to decrease the size of the tumor, confine it to a specific body region, and facilitate its resection. Regarding the role of postoperative radiotherapy, a meta-analysis including 11 clinical trials has shown no advantage for this therapeutic approach and its derivatives [46]. Metallic nanoparticles have been proved to enhance radiotherapy effects, introducing a new type of approach for this treatment. The use of metallic nanoparticles for photothermal ablation leads to local cell death and tumor remission, increasing the efficacy of radiotherapy at lower doses [47]. However, for locally advanced disease—IIIA and IIIB—radiotherapy is not enough. Common treatments at these stages include chemotherapy and surgery-combined approaches, depending on the patient’s conditions [48]. Cisplatin-based chemotherapy is the preferred approach, although new drugs are being investigated.

### 2.2. Chemotherapy

Chemotherapy has largely demonstrated to be effective at all stages of NSCLC. For early stages, chemotherapy is still offered after complete resection, although there is uncertainty about a clear benefit to patients in stage I [49,50]. Chemotherapeutic drugs kill cancer cells but they also present offset cytotoxicity [43]. However, the main drawback of chemotherapy is the development of resistance mechanisms. At this point, numerous studies have demonstrated the importance of autophagy and apoptosis, related to the very common p53 mutation in NSCLC [51,52]. Autophagy, a "self-eating" process, has a dual role in the regulation of apoptosis in lung cancer. Its activation is a control mechanism for the suppression of tumorigenesis, but its abnormal activation—maintained by conditions of and signals from the tumor environment—protects the tumor cells, contributing to resistance. In this regard, the development of nanotherapies for the modulation of autophagy has turned out to be a complementary strategy to chemotherapy, of important clinical relevance. In particular, metallic nanoparticles are effective in this application [53]. Interestingly, Ke et al. (2017) [54] evaluated the effect of gold nanoparticles in combination with anti-tumor therapies for modulating the expression of proteins related to the processes of apoptosis and autophagy. Promising results encourage the investigation of new approaches in this field.

Despite its toxicity profile and short-term effectiveness, chemotherapy remains a first-line treatment for NSCLC. In general, six categories of substances used in chemotherapy are distinguished: (a) alkylating agents; (b) anti-metabolites competing with the normal precursors of RNA and DNA; (c) antibiotics, inhibiting enzymes involved in DNA replication; (d) topoisomerase inhibitors, avoiding DNA replication and transcription, and (e) mitotic inhibitors (Table 3). Currently, there are FDA-approved drugs within each of these categories, but nanotechnology seems to improve their effects, overcoming their intrinsic conventional limits (Table 4). Here, we will discuss challenges posed by mitotic inhibitors and alkylating and anti-metabolite agents, wherein significant enhancements have been noticed thanks to new nano-formulations.

#### 2.2.1. Mitotic Inhibitors

Mitotic inhibitors are anti-cancer drugs preventing cell mitosis. Targeting mitosis is an approach based on the high proliferation rate of cancer cells compared to healthy cells, although off-site cytotoxicity is still considerable [57,58]. These types of treatments aim to destabilize or stabilize the microtubules—protein polymers that play a fundamental role during the final phase of mitosis. The destabilizing and stabilizing mitotic inhibitors act in different phases of mitosis. The former prevents chromosome alignment during metaphase, while the latter prevents cell division in later phases [57,58]. Stabilizing agents include paclitaxel, a first-generation drug that has been extensively studied and characterized. Although it continues to be a first-line treatment, paclitaxel has a very high toxicity profile. Its nonspecific activity affects healthy dividing cells and other cells such as neurons, where intracellular transport processes are linked to the activity of microtubules [59,60].

New forms of administration are being investigated to reduce the cytotoxicity and the required dose of paclitaxel. The most notable among them is the formulation of the drug with albumin, known as Abraxane^®^, forming nanoparticles around 130 nm in diameter. Abraxane^®^ has shown excellent results in terms of paclitaxel solubility and required dose reduction and considerable increases in overall response rate (ORR) in combination with carboplatin [59]. In addition to this formulation, the use of extracellular vesicles (EVs) has recently shown promising results in the encapsulation of antitumor drugs [60]. This type of vesicles, which includes exosomes, microvesicles, and apoptotic bodies, has advantages in terms of biocompatibility, immunogenicity, and cytotoxicity. The results obtained after firstly encapsulating doxorubicin—a topoisomerase inhibitor—in exosomes, have boosted the use of this type of DDS, creating high expectations [61]. Several studies indicate paclitaxel was delivered successfully to tumor cells via tumor and mesenchymal stem cell-derived exosomes [62,63,64]. Modifications of exosomes by incorporating signal peptides to their surface improve the biodistribution of the drug and hinder the development of drug resistance mechanisms [60].

#### 2.2.2. Alkylating Agents

Alkylating agents performed a significant role in the development of chemotherapy. In the 1970s, several substances presenting this activity were identified and used as DNA cross-linkers, preventing the replication of tumorigenic cells. Examples of these drugs are Cisplatin, Mitomycin-C, Ifosfamide, Vindesine, Vinblastine, and Etoposide. However, despite the promising initial results, tumors were observed to develop resistance after 2–3 months of treatment [65]. Consequently, drug combinations have been studied to prolong the therapeutic effect. Most studies combine cisplatin along with other alkylating or effective drugs against some characteristic patient mutations. Caution must be exercised regarding NSCLC, as it has shown 68% and 63% resistance against carboplatin and cisplatin and a similar percentage spectrum against other alkaloids [66]. Various studies propose the intracellular accumulation of drugs or the increase in the DNA repair capacity of tumor cells as possible resistance mechanisms [67]. Consequently, an increasing number of clinical studies aims to identify those subgroups of patients who may benefit from targeted agents due to characteristic mutations such as those of the epidermal growth factor receptor (EGFR) [38]. The need to develop a strategy that allows maintaining the effectiveness of a drug over time is necessary for a long-term NSCLC treatment.

In addition to the development of tumor resistance, the high cytotoxicity of these drugs has prompted numerous studies to determine whether the derived benefits were sufficient. The systemic administration of chemotherapy drugs involves their internalization by healthy cells [31,34,68]. It is widely accepted that the high proliferation rate of cancer cells makes them more susceptible to drug toxicity, since they cannot repair DNA while they proliferate [65,66]. However, the patient’s quality of life is decreased by chemotherapy’s collateral cytotoxicity. Currently, the effectiveness of chemotherapy, together with nanotechnology, can provide the answer to the historical challenges that the former has faced. Polymeric micelles were the first nano-vehicles to be studied and approved to improve drug biodistribution and reduce the cytotoxicity of chemotherapy [67]. There are some drugs in the clinical phase and others already approved that encapsulate paclitaxel or other taxanes to attack tumors. Genexol-PM, currently in phase II in the USA and already approved in Europe and South Korea, encapsulates paclitaxel in poly(ethylene glycol)–b-poly(lactic acid) (PEG–b-PLA) copolymer micelles. The results obtained so far show a favorable overall response rate from 37.7% to 46.5% and also confirm that the encapsulation strategy is effective to reduce collateral cytotoxicity, thanks to the absence of surfactants, such as Cremophor EL, that solubilize this type of highly hydrophobic drugs [69,70]. NK-4016, NC-6004, and NC-6300 are another type of polymeric nanoparticles that allow dissolving platinum or derivatives, increasing its solubility and decreasing its systemic toxicity [71,72,73].

#### 2.2.3. Antimetabolite Drugs

Antimetabolites are molecules preventing the synthesis of DNA and RNA by binding and stabilizing the enzymes involved in this process or replacing nucleotides in the nuclei acid growing chain. Therefore, their activity is limited to the DNA replication phase during cell proliferation. This restriction limits their efficiency, which is not proportional to the administered concentration, since the drugs only affect cells in the S and M phases of mitosis [43,67,74]. Regardless of the increase in drug doses, a plateau effect is early achieved at low concentrations [75]. Also, due to their lack of specificity and high off-target cytotoxicity, it is necessary to carefully determine the dose of drug administered to the patient. Several molecules with this activity are currently available (antifolates, fluoropyrimidines, deoxynucleotide analogs, and thiopurines), although a reduced number of them is used for treating NSCLC [67,74,76].

For several years, their application as a cancer treatment did not seem to be effective for NSCLC, but Pemetrexed—a multitargeted antifolate—was proved to be a very active agent [74,77]. Randomized trials in phase III, comparing Pemetrexed and Doxorubicin, antimetabolite and inhibitor drugs, respectively, showed similar clinical efficacy but considerably fewer side effects for Pemetrexed [78]. Subsequently, the use of other drugs such as methotrexate and gemcitabine, extensively investigated for the treatment of other cancers, was approved [74,79].

Fluoropyrimidines and derivatives have recently gained interest for the treatment of NSCLC [80,81,82]. Among them, 5-Fluorouracil (5-FU) has shown promising results in the treatment of breast and colorectal cancer but is not routinely administered for NSCLC treatment [82,83,84]. Several clinical trials are now ongoing to evaluate the treatment with 5-FU and derivatives for advanced NSCLC (NCT01658813, NCT02855125, NCT03421353, NCT02009449, NCT00019513). The drug 5-FU is a molecule derived from pyridine that, after cell internalization, metabolizes to 5-fluoro-2’-deoxyuridine monophosphate (FdUMP), 5-fluoro-2’-deoxyuridine triphosphate (FdUTP), and triphosphate 5-fluorouridine (FUTP). Each of the above metabolites plays an anti-tumor role; FdUMP inhibits the function of the enzyme thymidylate synthase (TS), while FdUTP and FUTP join the growing chain of DNA or RNA, leading to cellular damage.

**Figure 3:**
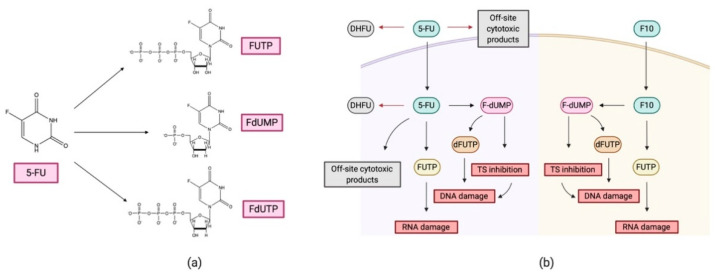
The anti-metabolite drug 5-fluorouracil (5-FU)**.** (**a**) Catabolism of 5-FU to triphosphate 5-fluorouridine (FUTP), 5-fluoro-2’-deoxyuridine monophosphate (FdUMP), and 5-fluoro-2’-deoxyuridine triphosphate (FdUTP). (**b**) Schematic of uptake and important metabolites of 5-FU and F10 mechanisms interfering with DNA and RNA replication.

The application of 5-FU for NSCLC treatment has evolved to the use of derived oligomers [85,86]. This strategy allows avoiding the resistance mechanisms that the tumor generates against 5-FU activity (Figure 3). Tumor cells respond to the administration of 5-FU by increasing the production of deoxyribonucleotides that compete with 5-FU metabolites or by increasing the catabolism of 5-FU to other forms tha are not harmful to the tumorigenic cells [82]. The use of oligomers such as F10—a DNA polymer containing FdUMP monomers—in combination with delivery nanosystems is highly promising for the treatment of NSCLC [84,86].

### 2.3. Molecular Targeted Therapies: Tyrosine Kinase Inhibitors (TKIs)

This type of treatment is sometimes considered as chemotherapy, but although the two types of treatment have similar targets, TKIs depend on the existence of mutations in specific receptors. Limited effectiveness of chemotherapy and remarkable advances in the field of genetics have encouraged the development of new strategies for the treatment of NSCLC. Chemotherapy was initially administered as standard of care; however, it was later understood that NSCLC is a molecularly heterogeneous disease [87]. NSCLC subclassification into different groups according to the presence of driver mutations, i.e., mutations that are essential for the proliferation or survival of tumor cells, inspired the design of a new class of anti-tumor drugs. The activity of these mutations is decisive for tumor development and leads to “oncogene addiction”, which highlights the essential role of these oncogenes for the tumor, even in the absence of mechanisms inhibiting tumor suppressors [75,88].

**Figure 4:**
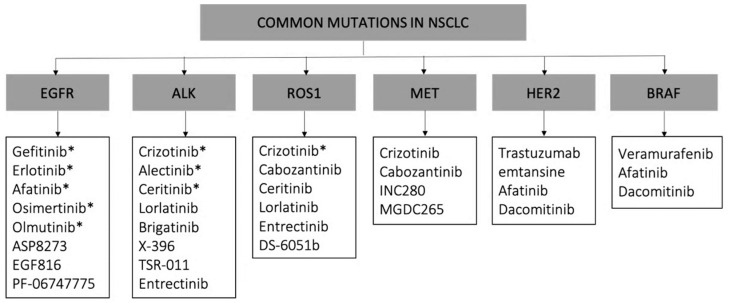
Available molecular targeted drugs for NSCLC [87]. * FDA-approved drugs for NSCLC.

The first evidence for the future of TKIs was the approval of gefitinib, an EGFR inhibitor (Table 5). The mutation modifying the tyrosine kinase activity of this receptor is one of the most common in NSCLC, affecting the mechanisms of tumor proliferation, angiogenesis, motility, survival, and differentiation [89,90]. A random clinical trial of 200 patients proved the effectiveness of gefitinib against conventional chemotherapy as a first-line treatment, setting the beginning for the development of these anti-tumoral agents. Results showed significantly higher median overall survival in patients treated with gefitinib (30.5 months) and a higher response rate when compared to patients treated with chemotherapy. Other common oncogenes are KRAS (exclusive with EGFR), ALK, HER2, BRAF, and MET (Figure 4). The activity of vast majority of these oncogenes is related to the phosphorylation of tyrosine, serine, and threonine receptors, which are involved in complex cell division and growth pathways [75,87,91,92]. The identification of these mutations is of relevant clinical interest for the development of new effective drugs for specific genotypes of NSCLC patients. In addition to the above-mentioned oncogenes, there are gene rearrangements that, although less common, play important roles. This is the case of the RET oncogene. The RET rearrangement is only present in 1–2% of NSCLC patients but is closely related to the development of resistance mechanisms against TKIs [93]. The design of multi-kinase inhibitors with anti-RET activity drugs is of special interest since it allows prolonging the efficacy of these treatments [94]. Consequently, many of these inhibitors are considered as first-line treatments because they show greater efficacy and less off-target cytotoxicity than conventional chemotherapy. Despite this, the administration of these inhibitors is subject to chemical limitations. They are sparingly soluble molecules, small, and therefore easily permeable and very susceptible to binding to plasma proteins [95,96].

Most TKIs are administered orally, which considerably complicates reaching the desired target. For this reason, the use of nano drug delivery systems allows their administration through more appropriate routes [95,96]. Bakhtiary et al. (2017) [99] proposed the administration of erlotinib encapsulated in solid lipid nanoparticles through a dry powder inhaler. This type of nanocarrier improves the stability of hydrophobic and hydrophilic molecules, avoiding the use of organic solvents and enhancing the pharmacokinetic profiles of drugs [99]. Besides, encapsulation in nano-drug delivery systems is a remarkable step forward regarding drug administration and stability as well as treatment strategy. The development of combined therapies including TKIs and other immunosuppressive drugs allows attacking a tumor through multiple pathways. Thus, Han et al. (2018) [100] have shown that the PEG–PLA nanoparticles, approved by the FDA, are a great alternative to direct oral administration of drugs, since they allow to co-encapsulate drugs such as gefitinib and cyclosporin A and administer them to a tumor in a single vehicle. Recently, the use of antibody–drug conjugates (ADC) targeting oncogenes has significantly improved the specificity of TKIs, reducing their cytotoxicity. The benefits of coupling TK inhibitors to recombinant monoclonal antibodies (mAbs) directed against tumor-associated antigens rely on the high specificity of this mAbs. mAbs allows reducing considerably the maximal inhibitory concentration of drugs, improving their toxicity profile [101].

## 3. Emerging Treatments

Currently, the main treatments for NSCLC remain surgery, chemotherapy, and radiotherapy, but the strong limitations concerning their efficacy over time and their side effects have pushed research to new alternatives. Despite the incorporation of molecularly targeted therapies in combination with chemotherapy, the increase in NSCLC patients’ overall survival (OS) has achieved a plateau effect in the last years [43,102,103]. The use of immunotherapy, together with gene therapy, is expected to stimulate the production of the next generation of drugs, improving the overall responses in NSCLC patients.

### 3.1. Immunotherapies

**Figure 5:**
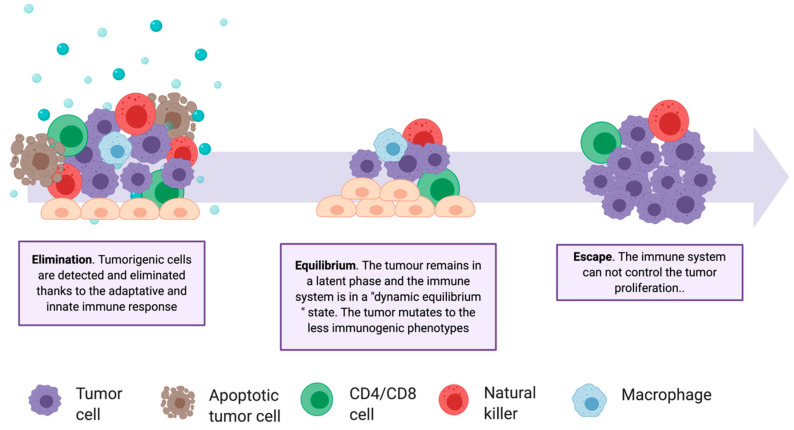
The complexity of the tumor–immune system relationship. Schematic representation of the three phases of the Immunoediting Theory and of the involved cell types. Tumor heterogenicity increases through the elimination phase, selecting the less immunogenic variants.

In recent years, immunotherapies have gained importance, as evidenced by the increase of related clinical studies [104,105]. These treatments stimulate the immune system in multiple ways and are personalized, being based on a patient’s genetic and epigenetic alterations. Historically, it has been considered that the immune system does not have, or has very little, response capacity against tumors. However, in 1950, Brunet and Farlane introduced the idea of immunosurveillance, proving that both the innate and the adaptive immune systems are capable of detecting a tumor and reacting to it in the early stages of its development [106,107,108]. Despite immunosurveillance, tumors continually develop resistance and defense mechanisms, creating a tumor immunosuppressive microenvironment (TIME) and escaping the action of natural killer cells, CD8^+^ T cells, CD4^+^ T cells, and macrophages (Table A2). The communication between the two environments—the tumor and the immune environments—is described by the immunoediting theory (Figure 5), which includes three stages: elimination, equilibrium, and escape [108]. Immunotherapies are mainly focused on the enhancement of the responsiveness of tumor-infiltrating immune cells during the elimination stage. Several approaches are investigated for this purpose, involving vaccine design, modification of immune cells, and inhibition of tumor evasion mechanisms.

#### 3.1.1. Immune Checkpoint Inhibitors

**Figure 6:**
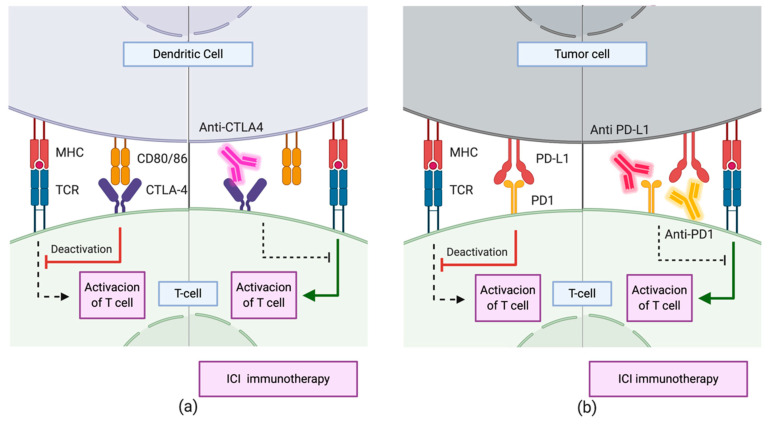
Immune checkpoint inhibitor immunotherapy through mechanisms involving (**a**) CTLA-4 and (**b**) PD1/PD-L1. During the priming phase, T cell activation requires two complementary signals: the engagement of the MHC complex to the T cell receptor (TCR) and the absence of interaction between CTLA-4 and CD80/86. Conversely, T cell activation will be strongly suppressed. Similarly, during the effector phase, the absence of interaction between PD1 and PD-L1 upregulates T cell activation.

Current immunotherapeutic strategies are mainly based on Immune Checkpoint Inhibitors (ICIs) [102,109]. These drugs target routes regulating the activation of the immune system through lymphocytes. The CTLA-4 and PD-1 immune checkpoint pathways have been extensively studied and characterized (Figure 6). Both inhibit T cell activation through different mechanisms and at different levels [110]. CTLA-4-dependent mechanism works in the early stages of immune activation, avoiding an overresponse from the immune system. Upon activation of T cells by antigen presentation, CTLA-4 expression occurs on the cell surface. This receptor interacts with CD80 and CD86 expressed on the membrane of Antigen-Presenting Cells (APC). The interaction inhibits the immune response and is enhanced by the tumor microenvironment (TME) through the release of different cytokines. Treatments with CTLA-4 checkpoint inhibitors consist mainly in the administration of antibodies with high recognition specificity for CTLA-4, preventing it from binding to CD80/86 ligands [109,110]. On the other hand, the PD-1/PD-L1 mechanism downregulates the immune response in late stages, during the effector phase of the innate–specific response of the immune system. The expression of PD-1 in the membrane of natural killer cells and the T/B cells occurs after its activation. The interaction of this protein with its PD-L1 and PD-L2 ligands downregulates the activity of T cells, avoiding autoimmune responses in healthy systems. However, in the TME, tumor cells either express these ligands on their surface or promote their expression by other immune cells through the segregation of factors such as IFN-γ The TME suppresses the immune response by limiting the activation, proliferation, survival, and effector functions of T cells. Thus, the administration of specific antibodies to PD-1 blocks the interaction of this protein with PD-L1 and PD-L2, avoiding the downregulation of this pathway. Other drugs inhibit it by sequestering PD-L1, which also inhibits the action of T cells. However, as the engagement of PD1 to PD-L2 is still possible, the downregulation of the pathway is limited, and as a result, the former strategy reduces the immune-related toxicity and the side effects of therapy.

The blockade of the CTLA-4 and PD1/PDL-1 mechanisms consists mainly in the administration of immunoglobulin G (IgG). Different agents are under clinical evaluation, and some of them, such as nivolumab and pembrolizumab, have been approved for the treatment of NSCLC [111,112]. The principal differences among them lie in the isotype of the administered IgG, as well as in their binding specificity, leading to differences in clinical activity [102]. Numerous clinical studies have evidenced that checkpoint inhibitors-based immunotherapy is effective as a second-line treatment or in advanced-stage patients, mostly in combination with chemotherapy, although pembrolizumab and atezolizumab are also administered as monotherapy [96,99,100] (Table 5). Efforts are currently focused on the performance of head-to-head studies that allow direct comparison between single agents and an agent in combination with chemotherapy [113]. Despite their potential, to date, clinical results indicate that ICIs only benefit a subset of patients and present low response rates [114]. Importantly, recent studies have evaluated the improvements of ICIs’ anti-tumoral effect when these molecules are combined with chemotherapeutic drugs, which can also modulate the immune activity. Preclinical studies on the combination of nanoparticle-mediated chemotherapy and immune checkpoint inhibitors have shown highly interesting results in murine tumor models. Kuai et al. (2017) have recently published the design and preparation of a chemotherapeutic drug delivery system consisting of high-density synthetic lipoprotein (sHDL) nanodiscs. This type of delivery platform allows the safe and effective release of different chemotherapeutic drugs and enhances the immune response by inhibiting the PD1/PD-L1 pathway [115,116].

In addition to the co-administration with chemotherapy, immune checkpoint inhibitors can be combined with drug-loaded nanoparticles [32,117]. One of the most promising emerging strategies in this field is the use of photodynamic and thermal nanoparticle-enhanced therapies [118]. Photothermal therapies (PTT), used to photosensitize and treat localized cancers, are minimally invasive and are based on the release of vibrational energy from nanomaterials to ablate cancer cells. The advantage of this type of combined therapy compared to checkpoint inhibitors is that they allow overcoming the adaptive immune evasion mechanisms of tumors. Ge et al. (2018) [119] proposed the use of iron oxide (Fe3O4 superparticles) based on already FDA-approved particles for magnetic resonance imaging (MRI), encapsulated in spheres of the FDA-approved copolymer mPEG–poly(lactide-co-glycolid) (PLGA). These Fe3O4 superparticles are triggered by near-infrared (NIR) light, producing thermal ablation and killing tumor cells. Besides, these magnetic nanoparticles are encapsulated together with the immune adjuvant Toll-like receptor 7 (TLR7), which stimulates a strong systemic antitumor immune response. This strategy, involving three FDA-approved components, combined with PD-L1 immune checkpoint inhibitors, was proved to directly destroy a tumor upon NIR irradiation and induce dendritic cell activation [119].

#### 3.1.2. Therapeutic Vaccines

**Figure 7:**
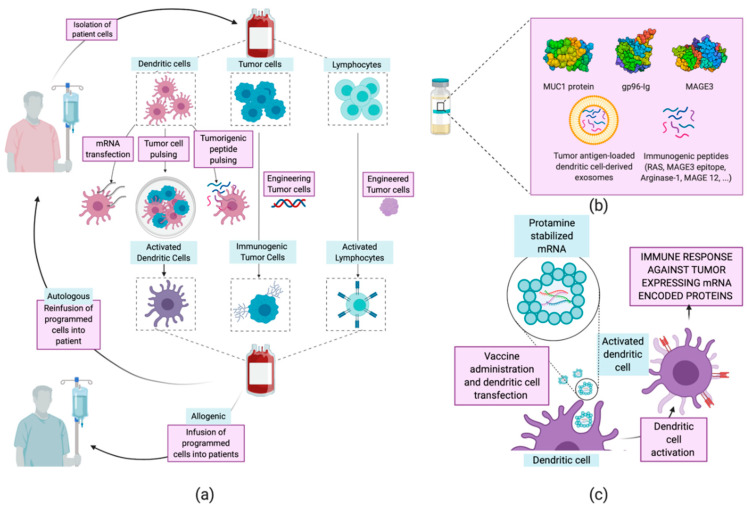
Current strategies for NSCLC cancer vaccines. (**a**) Whole-cell cancer vaccines. Dendritic, tumor, or lymphocyte cells are removed from the patient and modified ex vivo to increase their immune/immunogenic activity. Finally, they are delivered back to the donor patient—autologous therapy—or other patients—allogeneic therapy. (**b**) Protein- and peptide-based vaccines. Systematic administration of proteins or peptides previously identified as tumor antigens. (**c**) mRNA vaccines (RNActive^®^). Administration of five mRNAs, recognized for their immunogenic nature, stabilized by complexing with cationic proteins for the easy transfection of dendritic cells that will activate the immune response, entering the priming phase of T cells.

The term “vaccine” has been traditionally related to the treatment of infectious diseases, aiming at humoral immunity against pathogens. These types of vaccine have served as inspiration and have evolved into therapeutic vaccines [120]. The latter are designed to treat a disease by boosting the immune humoral and cellular—mainly T cell—response. The concept of a vaccine against cancer arises from the identification of mutated proteins, expressed aberrantly by tumor cells. These are identified as tumor-associated antigens (TAA) by the immune system and can be classified into expressed fetal antigens (normally absent in healthy adults) and overexpressed normal proteins. [120,121,122] Training the immune system to recognize and respond to these antigens is the working principle of therapeutic vaccines. Several vaccination strategies have been examined for the treatment of NSCLC, including whole-cell vaccines [123,124,125], protein- and peptide-based [126,127] vaccines, and mRNA vaccines [128,129] (Table 6 and Figure 7). Herein, we will focus on protein, peptide, and mRNA vaccines, as interesting advances have been achieved regarding their encapsulation using nanoparticles.

##### Protein and Peptide-Based Vaccines

The use of proteins or peptides for the treatment of cancer is one of the strategies first developed, in parallel with whole tumor cell vaccines. However, two main limitations explain the low response rate of this type of vaccine. First, cancer antigens show low immunogenicity on their own, requiring co-administration of adjuvants to stimulate the immune system [120,121]. Furthermore, the absence of proteins exclusively expressed in cancer cells increases the risk of triggering autoimmune responses. Together with safety issues, antigen proteins present complex glycosylation patterns and are difficult to purify, which hinders the from-bench-to-industry process. Thus, the use of peptides allows improving the stability, selectivity, and reduction of unwanted immune responses concerning the use of the complete protein. Despite this, in recent years several protein vaccines have been consolidated as effective treatments against NSCLC. The melanoma-associated antigen A3 (MAGE-A3) is an antigen almost exclusively expressed in various types of tumor cells. The administration of MAGE-A3 together with immune response-enhancing adjuvants has shown positive results, although not clinically relevant [130]. Similarly, the TG4010 vaccine uses the mucinous glycoprotein-1 protein (MUC1), another well-described tumor-associated protein, as an antigen. Although the vaccine based in the entire MUC1 protein did not obtain notable positive results in various clinical trials [131], the use of a 25-aminoacid MUC1 peptide showed remarkable positive outcomes [132,133]. Encapsulation of peptides in lipid particles allows overcoming limitations regarding peptide stabilization and protection and also improving the uptake by APCs. Furthermore, these types of carrier can be decorated with immune potentiators such as adjuvants or immune cell-targeting ligands [116,134].

##### mRNA Vaccines

mRNA vaccines appeared in the early 1990s after testing the expression of proteins from injected mRna [135]. At first, research on vaccines with genetic material focused mainly on DNA. The reason was the high stability of DNA when compared to RNA. However, the appearance of drug delivery nanosystems and the safety of mRNA in terms of mutagenicity and ease of internalization tipped the scale in favor of mRNA [134,136]. The activation of the immune response through cellular transfection with mRNA may be obtained through different approaches. Generally, the use of mRNA—encoding for one or more tumor-associated antigens—for the transfection of APCs trains them to recognize the encoded antigens and activate the humoral and cellular immune responses [137]. The safety of this type of vaccine and the conclusions obtained so far explain the number of emerging clinical trials (Table 6). RNActive^®^ CV9201 is one of the most promising vaccines available. It is composed of a mixture of five NSCLC-associated antigens which activate the immune response after the extraction of dendritic cells, their transfection, and subsequent delivery to the patient or after the direct administration of mRNA [136]. A phase Ib clinical trial of this vaccine proved a detectable immune response in over 65% of the patients. The strength of the induced response was variable, but 48% of the patients showed antigen-specific humoral responses [128]. RNActive^®^ CV9201 is based on the complexation of mRNA with protamine, a cationic protein with a great ability to complex negative molecules and facilitate their cellular internalization. This type of protein-based nanoparticles has shown promising results in in vivo tests, stimulating the adaptive immune response [138]. Nevertheless, it is worth noting that, being an autologous treatment, its application by healthcare systems is not affordable yet. Other studies revealed the promising use of EVs, such as exosomes, in developing cancer vaccines [60]. These vesicles allow both direct release of mRNA-based antigens to induce the immune response and in vitro co-culture of dendritic cells with antigen-loaded exosomes for maturation and subsequent injection into patients [139]. Furthermore, these vesicles are engineered through membrane decoration with viral fusion proteins or ligands for Toll-like receptors to enhance the immunogenicity of vaccines [60].

### 3.2. Modulating Gene Therapy

The main difficulty regarding the development of cancer treatments is the inherent and acquired drug resistance of tumors [140]. This remains the biggest obstacle to conventional treatments such as chemotherapy, reducing their short-term efficacy. Modulating gene therapies have gained interest in recent years to sensitize tumor cells against drugs. One of the most promising approaches is the use of RNA to silence the expression of certain proteins involved in tumor resistance. Transfection of tumor cells with silencing (si)RNA or long-non-coding (lnc)RNA related to tumor abnormalities has shown positive results [141,142,143]. Notably, delivery of siRNA as a gene knockdown strategy for the inhibition of the expression of certain genes related to apoptosis and cell proliferation has been tested as a sensitizing therapy by our group. One well-studied mechanism is the Target of Rapamycin (mTOR) pathway, which regulates cell proliferation and metabolism through the inhibition of apoptosis [48]. Previous studies have proved that siRNA encapsulation in polyplexes allows high transfection rates due to their highly positive charge. This nano drug delivery system presents an excellent endosomal escape capacity, ensuring the cytosolic delivery of siRNA [37,144,145,146].

In addition to sensitizing tumor cells to increase treatment efficacy and prevent tumor resistance mechanisms, it is necessary to control the metastatic potential and progression of lung cancer. One of the most relevant processes in NSCLC is epithelial–mesenchymal transition (EMT). EMT refers to the conversion of epithelial cells to mesenchymal cells by losing adhesion and gaining regeneration and differentiation capabilities [147]. This mechanism increases the metastatic and evolution potential of tumors in cancer patients. Thus, it is necessary to regulate EMT. The administration of micro (mi)RNA—single-stranded short non-coding RNA—allows regulating gene expression by the binding of miRNA to the end of the 3’ untranslated region (UTR) of the target mRNA [148]. As a result, the loss of markers and proteins characteristic of epithelial cells is avoided. Also other types of genetic material also can stop this conversion. For instance, Suresh et al. (2019) [149] designed antibody-conjugated gelatin nanoparticles that encapsulate siRNA for the inhibition of AXL expression, a kinase involved in various signaling pathways. In this way, they managed to reduce the activity of mTOR while reducing the expression of EMT proteins and increasing the tumor-suppressive activity of the p53 pathway.

Apart from silencing the expression of specific proteins, genome repair of lung cancer cells has become a popular approach for NSCLC treatment. The application of the CRISPR/Cas9 technology allows gene editing and opens a wide range of possibilities [150]. This approach is based on the use of single-guide RNA-directed Cas9. This enzyme cleaves the DNA at the point of interest and allows its sequence to be modified, deleted, or replaced. Consequently, the CRISPR/Cas9 system can be ubiquitously applied to knock out oncogenes and study tumor-suppressor genes and resistance-related genes [151].

## 4. Conclusions

**Figure 8:**
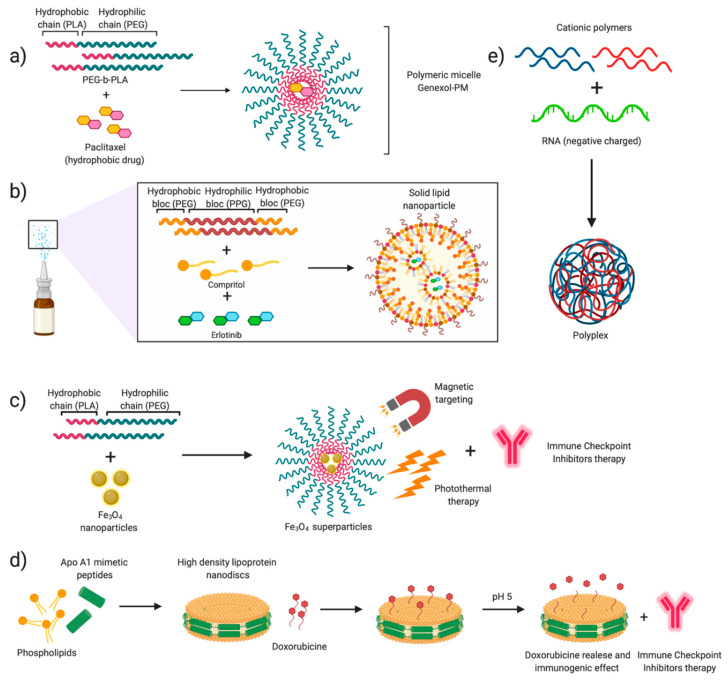
Nanotechnology for NSCLC. Summary of the mentioned examples describing different encapsulation systems. (**a**) Poly(ethylene glycol)–b-poly(lactic acid) (PLA–b-PEG) nanoparticles encapsulating Paclitaxel (Genexol-PM^®^) (NCT01023347, NCT01770795). (**b**) Solid–lipid nanoparticle formation for Paclitaxel encapsulation [99]. (**c**) Supernanoparticles for photothermal therapy in combination with Immune Checkpoint Inhibitors (ICIs) immunotherapy [119]. (**d**) Assembly of high-density-lipoprotein nanodiscs for doxorubicin delivery [115]. (**e**) Encapsulation of RNA in polyplexes, formed by cationic polymers [144,145,146].

In summary, the number of available treatments for NSCLC continues to expand, driven by the improvements introduced by nanotechnology. Conventional therapies have improved in terms of toxicity and clinical outcomes, thanks to their combination with nanomedicine. Nanodelivery systems aim to concentrate drugs in relevant cell populations and control drug release, improving their long-term effects. Furthermore, emerging immunotherapies show promising results for cancer treatment in the early stages. Novel therapeutic treatments based on siRNA, mRNA, and gene editing are the most encouraging cancer strategies. The perfect integration of these therapies with the versatility of nanotechnology (Figure 8) and their parallel growth suggest that the impact of these treatments on patient lives will be evident in a non-distant future. However, nanotechnology has to develop further. Our limited knowledge about the interactions between nanoparticles and biomolecules makes it difficult to fully understand the mechanisms involved and, therefore, to improve treatment design. Furthermore, research is required to describe the phenomenon of tumor nanoparticle permeability, as the current paradigm regarding EPR is being strongly questioned.

## Figures and Tables

**Table 1:** Subclassification of histological heterogeneity of Non-Small Cell Lung Cancer (NSCLC).

NSCLC Subtype	Percentage (% Over NSCLC Cases)	Characteristics	Ref.
Adenocarcinoma	40	Presents frequent histologic heterogeneity. Mainly affects the outer edges of the lung.	[10,19]
Squamous cell carcinoma	30	Centrally located in the larger bronchi of the lung. The incidence is linked with smoking more than for other NSCLC cancers.	[10,19]
Large cell carcinoma	10	Non-differentiated type of lung cancer that lacks the architecture of squamous or glandular differentiation. It usually affects the peripherical part of the lung.	[10,19]
Adenosquamous carcinoma	<5	It is a subtype presenting components of both adenocarcinoma and squamous cell carcinoma.	[10,19,20]
Sarcomatoid carcinoma	<1	Centrally located in the larger bronchi of the lung and the peripherical part of the lung. Hard to diagnose due to its unclear characteristics which are common to other cancer subtypes.	[10,19]

**Table 2:** Staging of NSCLC according to the International Association for the Study of Lung Cancer [21].

Stage	Tumor	Lymph Node	Metastasis
Occult carcinoma	TX	N0	M0
Stage 0	Tis	N0	M0
Stage IA	T1mi-c	N0	M0
Stage IB	T2a	N0	M0
Stage IIA	T2b	N0–N1	M0
Stage IIB	T1, T2, T3	N0–N1	M0
Stage IIIA	T1–T4	N0–N2	M0
Stage IIIB	T1a–T4	N2–N3	M0
Stage IVA	Any T	Any N	M1a–b
Stage IVB	Any T	Any N	M1c

**Table 3:** Food and Drug Administration (FDA)-approved chemotherapy drugs for the treatment of NSCLC. Sources, FDA and National Cancer Institute databases [55,56].

Generic Name (Brand Name)	Mechanism of Action
Carboplatin (Paraplatin^®^)	Alkylating agent
Docetaxel (Taxotere^®^)	Mitotic inhibitor
Doxorubicin Hydrochloride (Adryamycin^®^, Rubex^®^)	Topoisomerase inhibitor
Gemcitabine Hydrochloride (Gemzar^®^)	Antimetabolite
Lurtotecan (OSI-211)	Topoisomerase inhibitor
Mechlorethamine Hydrochloride (Mustargen^®^)	Alkylating agent
Methotrexate (TrexallTM, Rheumatrex^®^)	Antimetabolite
Paclitaxel (Taxol^®^)	Mitotic inhibitor
Paclitaxel–Albumin-stabilized Nanoparticle Formulation (Abraxane^®^)	Mitotic inhibitor
Pemetrexed Disodium (Alimta^®^)	Antimetabolite
Vinorelbine Tartrate (Navelbine^®^)	Tubuline-binding agent

**Table 4:** Active and completed nanomedicine-based chemotherapeutic drugs clinical trials.

Drug	Nano Delivery System	NSCLC Stage	Phase	Clinical Trial
**Doxorubicin Hydrochloride (Adryamycin** ^**®**^ **, Rubex ** ^**®**^ **)**	Pegylated Liposome	IIIB–IV	II	NCT01051362
	Aerosolized Liposome	IIIB	I	NCT00020124
**Paclitaxel**	Liposome	IIIB–IV	IV	NCT02996214
	Polymeric micelle (Genexol-PM^®^)	IV	II	NCT01023347
				NCT01770795
**Camptothecin**	Aerosolized Liposome	IIIB–IV	Pre-clinical	NCT00277082
**Lurtotecan**	Liposome	IIIB	I	NCT00006036

**Table 5:** Anti-checkpoint inhibitor drugs for NSCLC. * FDA-approved anti-checkpoint inhibitors.

Generic Name (Brand Name)	Mechanism	Ref.
Atezolizumab (Tecentriq^®^) *	PD-L1	[55,56]
Durvalumab (Imfinzi^®^) *	PD-L1	[55,56]
Nivolumab (Opdivo^®^) *	PD1	[55,56]
Pembrolizumab (Keytruda^®^) *	PD1	[55,56]
Ipilimumab (Yervoy^®^)	CTLA-4	[97,98]

**Table 6:** Relevant clinical trials evaluating cancer vaccines for NSCLC. *N.S.: Not Specified

Vaccine	Components (Brand/Clinical Trial Name)	NSCLC Stage	Clinical Study Phase	Clinical Trial
**Cellular vaccine**	Allogenic tumoral cells (1650-G)	I–II	II	NCT00654030, NCT00601796
	Autologous engineered dendritic cells (MIDRIX4-LUNG)	III	I	NCT04082182
	Autologous mRNA/DNA transfected dendritic cells (MIDRIXNEO-LUNG)	III–IV	I	NCT04078269
	Allogenic mRNA-transfected dendritic cells (AST-VAC2)	III–IV	I	NCT03371485
	Allogenic engineered dendritic cells irradiated with seven active agents (NY-ESO-1, MAGE C1, 4MAGE C2, TPGB, Survivn, MUC1, Melan-A antigen (PDC*lung01)	N.S.	I–II	NCT03970746
	Autologous dendritic cells pulsed with allogenic tumor cells	III	II	NCT00103116
	Allogenic whole tumor cells (Lucanix ^®^)	III–IV	III	NCT00676507, NCT01058785
	Autologous dendritic cells pulsed with allogenic tumor cells (MelCancerVac^®^)	III–IV	II	NCT00442754
	Autologous dendritic cells pulsed with p53 peptide	III	II	NCT00019929
	Engineered autologous killed tumor cells	IV	I–II	NCT01159288, NCT02439450
	Allogeneic CD4+ memory Th1-like T-cells (Allostim^®^)	II–IV	I–II	NCT01065441
	Autologous dendritic cells pulsed with allogenic tumor cells (DVAC/LuCa)	IV	I–II	NCT02470468
	Allogenic lymphocytes	I–IV	I	NCT00161187
**Protein vaccine**	MUC1	III	I–II	NCT01720836, NCT03353675, NCT00415818 NCT03623750
	Heat shock protein (gp96-Ig)	III–IV	I	NCT00503568
	Tumor antigen-loaded dendritic cell-derived exosomes	III–IV	II	NCT01159288
	Anti-idiotype vaccine	IIA–III	II	NCT00006470
	Recombinant PRAME protein	I–IIIA	II	NCT01853878
**Peptide vaccine**	IDO peptide	III–IV	I	NCT01219348
	HLA-A*0201 restricted 9-mer epitopes (Vx001)	IV	II	NCT01935154
	Short lived proteins (SLiPs) and defective ribosomal products (DRiPs)	III–IV	I	NCT00850785, NCT01909752
	Synthetic peptides encoding hTERT (UV1)	III	I–II	NCT01789099
	MUC1 peptide (Tecemotide/L-BLP25/Stimuvax^®^)	III	III	NCT00409188, NCT00960115, NCT00157196, NCT00828009, NCT00157209
	UCP2 and UCP4 (telomerase derived peptides)	III	I–II	NCT02818426
	Epitope Peptide Restricted to HLA-A*02	III–IV	I	NCT01069640, NCT01069575
	GV1001 (Synthetic peptides encoding hTERT)	III	N.E. (already approved in Korea for pancreatic cancer)	NCT00509457
	(MAGE3 epitope) (Astuprotimut-R (GSK-249553))	IB–II	II	NCT00290355
	Wilms tumor 1 (WT1) analog peptide (DSP-7888)	III–IV	I	NCT03715985
	Peptides derived from a patient’s tumor individual neo-antigens (NeoPepVac, GRT-C901 and GRT-R902, GEN-009, NEO-PV-01)	III–IV	I	NCT03715985, NCT03639714, NCT03794128, NCT03953235, NCT03633110, NCT02897765, NCT03380871
	Tedopi^®^ (OSE2101)	III–IV	III	NCT02654587
	RAS peptide	II–IV	I–II	NCT00019006, NCT00019331, NCT00003125
	Arginase-1 peptide	Generic	I	NCT03689192
	YE-NEO-001 Neoepitope yeast vaccine (YE-NEO-001)	Generic	I	NCT03552718
	MAGE-12 peptide	IV	I	NCT00020267
	Patient specific neoepitopes			
**mRNA vaccine**	NY-ESO-1, MAGE C1, 4MAGE C2, TPGB, Survivn, MUC1 (RNActive^®^)	III–IV	I–II	NCT03164772, NCT00923312
	KRAS gene vaccine V941 (mRNA-5671)	III–IV	I	NCT03948763
	Personalized vaccine against patient’s mutations (RO7198457)	III–IV	I	NCT03289962
**DNA vaccine**	NY-ESO-1 plasmid DNA (pPJV7611) to increase immunogenicity of tumor cells	III–IV	I–II	NCT00199849
	Plasmid encoding neoepitopes (VB10.NEO)	III–IV	I–II	NCT03548467

## Appendix A

**Table A1:** Imaging techniques for NSCLC diagnosis.

**Positron Emission Tomography (PET)**. Detects pairs of gamma rays emitted by a radioligand previously introduced in the body after the combination of the introduced positrons with electrons from the patients’ body. Thus, it requires the use of contrast agents conjugated to a biologically relevant molecule, involved in the disease to be detected [15,35,152]. **Computed Tomography (CT).** Measures the attenuation of X rays emitted after interacting with tissues. It may require contrast agents if the difference of attenuation after interaction with different tissues of interest is not evident. After the acquisition, cross-sectional images are obtained using tomographic reconstruction [15,35,152]. **Magnetic Resonance Imaging (MRI).** Measures changes in the nuclear momentum of atoms while applying an external magnetic field. Hydrogen atoms are usually evaluated due to their simplicity and abundance in the human body. The relaxing times of H atoms in different tissues are measured to obtain images [15,35,152]. Does not require contrast agents. **Bronchoscopy.** Imaging technique consisting of the use of flexible bronchoscopy, including a camera, to visualize the inside of the airways for diagnostic and therapeutic purposes [22,35,153]. **Mediastinoscopy.** Small incision in the center of the thoracic cavity to section a small part of the tissue for biopsy purposes [22,35,153]. **Bronchoalveolar lavage (BAL).** Introduction of a measured volume of fluid in the lungs through the appropriate airways for further examination [22,35,153].

## Appendix B

**Table A2:** Cells involved in the immune response.

**Dendritic cells (DC).** These are Antigen-Presenting Cells (APC), infiltrating in the tumor microenvironment and capturing antigens. They play an important role in both the adaptative and the innate immune responses, activating naïve T lymphocytes in secondary lymphoid organs [154,155]. **Natural Killers (NK).** They accumulate at the tumor site and are important secretors of IFN-γ. When activated, NK eliminate tumor cells independently of tumor antigen exposure. Their activity is regulated by activating and inhibitory signals released by normal and abnormal cells [156,157]. **CD8^+^ T cells.** Activated T cells recognizing antigens presented by MHC class I molecules found on all nucleated cells. The recognition of these antigens stimulates the release of cytokines and cytotoxic granules, destroying infected cells via Fas/FasL interactions [158]. **CD4^+^ T cells**. Activated T cells recognizing antigens presented by MHC class II molecules in APC cells. The recognition of these antigens stimulates the adaptative immune response through the maturation to T helper (Th)17, Th1, or Th2 cells [159]. **Macrophages.** Specialized cells involved in the detection and phagocytosis of bacteria and non-self-recognized elements. They are also APC cells and important cytokines releasers [160,161].
